# UNCOVERING THE HIDDEN WORKFORCE: A STUDY OF DIRECT CARE WORKERS IN ASSISTED LIVING HOMES

**DOI:** 10.1093/geroni/igad104.1725

**Published:** 2023-12-21

**Authors:** Christina Wyles, Kimberly Shea

**Affiliations:** Roudebush VA Medical Center, Indianapolis, Indiana, United States; College of Nursing, The University of Arizona, Tucson, Arizona, United States

## Abstract

A growing number of 4.5 million U.S. direct care workers (DCWs) provide hands-on care to older adult residents in assisted living homes (ALHs) -- the smallest type of assisted living residences that require employment of DCWs. These homes typically have capacity for 8-10 residents, and they no longer resemble the original non-medical, home-like environment. With an aging population, increasing complexity of residents’ medical and functional needs, and increasing closure of nursing homes, demand for DCWs in ALHs continues to grow. Better information on this growing workforce segment, federal oversight, and consistent licensure are needed. The few available studies indicate high resident acuity and quality of care issues. To address this knowledge gap, a study explored and described the learning interests and needs and technology use of ALH DCWs. Guided by a human factors framework and an interpersonal perspective, DCWs (N = 14/ median age 41) were interviewed one-on-one using semi-structured questions. Data analysis involved qualitative content analysis of transcribed taped interviews. Findings revealed DCWs are interested in work-related topics and acknowledge the complexity of their residents. They use various health-related technologies in the care they provide, but need more access to educational opportunities that accommodate their long work hours and low salaries. The study also noted the expanded scope of practice, high risk of injury, and underrepresentation of DCWs in the literature. This study highlights learning and technology needs of DCWs in ALHs, emphasizing the need for more recognition and support for this vital segment of the long-term care workforce.

